# Can a dentin bonding agent prevent color change in regenerative endodontic procedures? An in vitro evaluation

**DOI:** 10.1590/0103-6440202405550

**Published:** 2024-05-10

**Authors:** Eduardo Trota Chaves, Laura Lourenço Morel, Fernanda Geraldo Pappen, Flávio Fernando Demarco, Luciane Geanini Pena Santos

**Affiliations:** 1 Programa de Pós-Graduação em Odontologia, Universidade Federal de Pelotas (UFPel), Pelotas- RS- Brasil.; 2 Programa de Pós-graduação em Odontologia, Universidade Estadual de Campinas (UNICAMP), Campinas- SP- Brasil.

**Keywords:** dental pulp, regenerative endodontics, tooth discoloration, triple antibiotic paste

## Abstract

This in vitro study aimed to determine the efficacy of dentin bonding agents in preventing color changes following Regenerative Endodontic Procedures. One hundred twenty bovine incisors were endodontically prepared and randomly assigned to a two main factors design: application of a dentin bonding agent (Scotchbond Adper, 3M ESPE, St Paul, MN, USA) in the pulp chamber (Group 1, n=60) versus no bonding intervention (Group 2, n=60), and five levels of intracanal medication (n=12/subgroup): Triple antibiotic paste (TAP), double antibiotic paste (DAB), calcium hydroxide (CH), modified triple antibiotic paste (TAPM), and Control (CTL). Color changes were measured over 28 days at multiple time points (1, 3, 7, 14, 21, and 28 days) using the CIEDE2000 formula to calculate the color difference (ΔE00) from baseline (T0). The ΔE00 quantifies the perceptible color difference between the initial and final tooth color, with lower values indicating less discoloration. The results were analyzed using repeated measures ANOVA-2 and post-hoc Holm-Sidak tests. The TAP subgroups, both with and without the bonding agent, exhibited the highest color variation. However, a pulp chamber seal with a bonding agent showed a protective effect against discoloration compared to no seal, even though complete prevention was not achieved. All groups demonstrated ΔE00 values beyond acceptable interpretation thresholds for clinical application, primarily driven by a reduction in lightness (L*) and a decrease in redness (a* value, shifting towards green). In conclusion, while the pulp chamber seal with a bonding agent mitigated TAP-induced discoloration, it did not eliminate it.

## Resumo

Este estudo in vitro avaliou adesivos dentinários na prevenção de alterações de cor após procedimentos endodônticos regenerativos. Cento e vinte incisivos bovinos foram preparados endodonticamente e aleatoriamente designados para um desenho com dois fatores principais: aplicação de agente adesivo (Scotchbond Adper, 3M ESPE, St Paul, MN, EUA) na câmara pulpar (Grupo 1, n=60) versus não intervenção adesiva (Grupo 2, n=60), e cinco níveis de medicação intracanal (n=12/subgrupo): pasta de triantibiótica (TAP), pasta diantibiótica (DAB), hidróxido de cálcio (CH), pasta triantibiótica modificada (TAPM) e Controle (CTL). Alterações cromáticas foram monitoradas por 28 dias em intervalos (1, 3, 7, 14, 21, e 28 dias), usando CIEDE2000 para calcular a diferença de cor (ΔE_00_) em relação a cor inicial. O ΔE_00_ quantifica a diferença entre a cor inicial e final do dente, com valores menores indicando menos descoloração. Os resultados foram analisados usando ANOVA-2 de medidas repetidas e teste posthoc de Holm-Sidak. Os grupos utilizando medicação TAP, com ou sem adesivo, possuíram as maiores variações cromática. Contudo, o uso do adesivo na câmara pulpar mostrou um efeito protetor contra descoloração, em comparação com a ausência do selamento adesivo, embora a prevenção completa não tenha sido alcançada. Todos os grupos demonstraram valores de ΔE_00_ além dos limiares aceitáveis para aplicação clínica, principalmente devido à redução na luminosidade (L*) e redução no vermelho (a*, deslocando-se em direção ao verde). Em conclusão, enquanto o selamento da câmara pulpar com um agente adesivo mitigou a descoloração induzida pelo TAP, mas não a eliminou completamente.

## Introduction

Pulp necrosis could challenge immature permanent teeth endodontic treatment, impairing dentinogenesis and preventing complete root formation ^1^. In these cases, the root remains underdeveloped in length and thickness, resulting in a fragile structure susceptible to fracture ^2^ ^,^ ^3^.

Regenerative Endodontic Procedure (REP) has been proposed as an alternative to conventional endodontic treatment ^4^. REP aims to provide a suitable environment for the dentinogenesis process continuation. This approach uses chemical treatment of root canals with minimal or no mechanical instrumentation ^3^ ^,^ ^5^. Subsequently, periapical tissues are stimulated for intracanal bleeding, establishing a blood clot and filling the root canal space ^6^. REP is based on the principles of tissue engineering, where the interaction of stem cells triad, framework, and morphogenetic proteins results in tissue neoformation ^5^ ^,^ ^6^.

The procedure itself presents a high level of complexity. The root canal system’s internal walls should be disinfected, free of bacteria from the necrotic process ^2^ ^,^ ^3^; at the same time, disinfection should not interfere with cell survival and proliferation. In a recent review, laboratory studies were mapped, and promisor results were found for REP, even in the presence of highly resistant bacteria, such as *E. faecalis* ^3^. Nevertheless, the development of this field of knowledge is still lacking, considering the reported collateral events, such as crown discoloration, the use of high cytotoxicity drugs, and the threatening drug removal from the dentinal walls ^2^ ^,^ ^3^ ^,^ ^4^ ^,^ ^5^.

Different antimicrobial pastes have been studied for adequate root dentin disinfection, an essential step of REP ^7^. Triple antibiotic paste (TAP) has been proposed to disinfect the root canal. Although the benefits of this medication, crown color changes can occur as a side effect ^4^, caused mainly by the minocycline present in its formulation ^8^. As an alternative, DAB (double antibiotic) paste, composed of two antibiotics (ciprofloxacin and metronidazole), has been indicated. Also, a "modified TAP" (TAPM), where minocycline is replaced with amoxicillin or cefaclor, has been recommended ^6^ ^,^ ^7^ ^,^ ^8^.

To minimize the color changes in the dental crown after REP, the seal of pulp chamber walls with dentin bonding agents can also be recommended ^9^. Bonding agents can obliterate the dentinal tubules opening, preventing or minimizing the color changes in coronal dentin. However, using adhesives to seal the pulp chamber can also affect the dental crown's or the restorative surfaces' shade ^10^. Then, this study aims to evaluate the influence of bonding agents on the color change of bovine-extracted teeth before REP’s procedures with different medications. It was hypothesized that the minocycline groups would perform more significant color changes. Also, it was hypothesized that the bonding agent seal could act as a protective factor against crown discoloration.

## Materials and methods

The estimation of our sample size was based on prior studies that investigated the color alteration in bovine incisors following various endodontic treatments. One such foundational study utilized a sample of 50 teeth (10 per group) ^11^, which provided a benchmark for the number of specimens that would likely allow for the detection of significant color changes with similar treatments.

In this context, we determined that ten specimens per group would be a logical starting point, given the analogous nature of our experimental design to that of the referenced study. To further account for any unforeseen loss of samples or experimental variance, particularly related to the sealing restoration at the apical third, we increased our sample size to twelve specimens per group. This conservative approach was chosen to ensure the stability and reliability of our study outcomes. The alpha level was set at the conventional 0.05, reflecting standard significance criteria in dental research.

The study's design involved two primary independent variables: ^1^ the application of a bonding agent to the pulp chamber walls and ^2^ the type of intracanal medication used. Each independent variable consisted of multiple levels as follows:

**Independent Variable 1: Pulp Chamber Sealing**

Level 1: Sealed with a dentin bonding agent (BA group)

Level 2: No dentin bonding agent used (non-BA group)

**Independent Variable 2: Intracanal Medication**

Level 1: Triple antibiotic paste (TAP)

Level 2: Modified triple antibiotic paste (TAPM)

Level 3: Double antibiotic paste (DAB)

Level 4: Calcium Hydroxide (CH)

Level 5: No medication (control)

The final sample was divided into ten groups, independent from each other but dependent on each group, once this is a repeated measures design study. One hundred and twenty bovine incisors, extracted for commercial reasons, were selected for the study. Samples with cracks or fractures were excluded. The teeth were rinsed with saline solution, followed by 1.5% sodium hypochlorite solution (NaOCl) and ultrasonic activation for 3 minutes for complete periodontal and pulp tissue dissolution. The samples were then kept in Timol solution until use ^11^.

### Samples’ preparation

To simulate the conditions of immature permanent teeth, the roots were sectioned 15 mm below the cementoenamel junction using a diamond disc on a high-speed handpiece under refrigeration. The 4 mm apical portion of the root canal was restored with resin composite (Z250XT, 3M ESPE, St Paul, MN, USA). After coronal access, the pulp tissue was removed using an Hedström #60 file (Dentsply-Maillefer), and the root canal was prepared using Gates Glidden drills (Dentsply-Maillefer) sizes # 3, #4, #5, and #6 ^12^. The root canals were irrigated with 1.5% NaOCl and 17% ethylenediaminetetraacetic acid (EDTA) (20 mL, for 5 minutes each and then dried using paper points. A cotton pellet was placed in the pulp chamber, and the coronal access was sealed with restorative glass ionomer (Riva Self Cure - SDI). During the procedures, samples were wrapped in gauze moistened in distilled water to avoid dehydration. After all, the samples were stored in 10mL of distilled water at 37ºC in individual flasks ^11^ ^,^ ^13^.

### Color Evaluation

The color assessment was performed using a digital spectrophotometer (Vita Easyshade®, Vident, Brea, CA, USA), previously calibrated according to the manufacturer’s instructions. The mean value of three measurements of each sample was registered. To standardize area and light conditions for color assessment, for each tooth, a custom silicone matrix was produced with impression material (Perfil®, Vigodent S/A, Rio de Janeiro, RJ, Brazil), covering the entire buccal tooth surface. A perforation in a compatible size with the spectrophotometer tip (±6 mm diameter) was made with a cutting-edge cylinder at the crown area ^11^ ^,^ ^13^.

The color assessment followed the CIE (Commission Internationale de l'Éclairage) terms, with the L*a*b* system, where “L*” represents light/brightness, “a*” green-red axle, and “b*” yellow-blue axle.

The first color assessment was performed one day after the specimens’ preparation (baseline). After removing specimens from distilled water, the excessive moisture of the external dental surface was removed with gauze.

### Group division

The specimens were randomly divided into two groups (n = 60). In group 1, the pulp chamber walls were sealed with a dentin bonding agent (BA group) (Scotchbond Adper, 3M ESPE, St Paula, MN, USA). In group 2, no dentin bonding agent was used (non-BA group). The groups were then subdivided (n= 12/group) as described below:

1a - TAP/BA: Bonded/Triple antibiotic paste;
1b - TAPM/BA: Bonded/Modified triple antibiotic paste;
1c - DAB/BA: Bonded/Double antibiotic paste;
1d - CH/BA: Bonded/Calcium Hydroxide;
1e - CTL/BA: Bonded/no-medication (positive control);
2a - TAP/non-BA: Non-bonded/Triple antibiotic paste;
2b - TAPM/non-BA: Non-bonded/Modified Triple antibiotic paste;
2c - DAB/non-BA: Non-bonded/Double antibiotic paste;
2d - CH/non-BA: Non-bonded/Calcium Hydroxide;
2e - CTL/non-BA: Non-bonded/no-medication (negative control)

A commercial 30-35% calcium hydroxide paste (Ultracal XS, Ultradent, South Jordan, UT, USA) was used. Antibiotic pastes were obtained from a manipulation pharmacy (Uso Indicado, Pelotas, RS, Brazil) at 0.1 mg/mL concentration and equal proportions of each antibiotic. All pastes were applied to the canals using a syringe/needle (22 G1). After placing the intracanal medication, a cotton pellet was placed in the pulp chamber, and the coronal access was sealed with restorative glass ionomer (Riva Self Cure - SDI). Samples were stored in a saline solution. After one, three, seven, 14, 21, and 28 days, the specimens had the excessive moisture removed, and color measurement was performed.

### Data and Statistical Analysis

Measurements of color alterations were performed from the values of L*, a*, and b* and confronted to T0 (Baseline), using the CIEDE2000 formula. The lower ∆E00 values, the smaller the difference between the initial and final tooth color over time. The data were analyzed by a two-way Analysis of Variance for Repeated Measures and a posthoc test of multiple Holm-Sidak comparisons at a significance level of 5% (p*<0.05). The ways were defined by the presence or absence of dentin bonding agent and the tested intracanal medication. Time was considered as the repeated measure. Once the present study presented a 2x5 design, ten experimental groups were evaluated. In this case, a normality test was unnecessary since parametric tests can give enough strength to assess interest outcomes ^14^. 

In our assessment of color changes, the interpretive parameters for acceptability and perceptibility were considered complementary assessments. Perceptibility refers to the minimum color difference that can be visually detected between two objects or the same object at different times. Acceptability represents the threshold at which color differences, though detectable, remain aesthetically acceptable to the observer. These concepts were defined by Paravina et al. 2015 ^15^, who established a 50:50% acceptability threshold where ΔE00 changes of 1.8 or less are considered an acceptable color match.

Expanding upon this foundational work, Paravina et al. 2019 ^16^ proposed a classification for color mismatches for ΔE00 changes beyond the acceptability threshold: changes from 1.8 to 3.6 were delineated as mismatch type A (moderately unacceptable), from 3.6 to 5.4 as mismatch type B (clearly unacceptable), and greater than 5.4 as mismatch type C (extremely unacceptable). Our study has adopted this classification system to assess the visual impact of color changes post-treatment, applying these meticulously developed criteria to evaluate the aesthetic outcomes of REP.

## Results

ΔE00 mean values of all evaluated groups were compared considering the variables treatment and experimental period. Significant differences were noticed in the interaction between these variables (p*=0.002), and they are presented in Table 1.

**Table 1:** Mean values of ΔE00 and standard deviation according to treatment and periods.

	TAP		TAPM		DAB		CH		Control	
	WOBA	BA	WOBA	BA	WOBA	BA	WOBA	BA	WOBA	BA
1d	3.04±1.8^aA^	3.48±1.7^aA^	3.48±2.2^aA^	2.55±2.9^aA^	3.83±2.6^aA^	3.04±2.2^aA^	2.71±1.8^aA^	3.31±2.0^aA^	2.43±1.4^aA^	2.49±1.0^aA^
3d	6.24±2.8^aA^	3.56±2.0^aAC^	3.89±1.8^aAC^	3.07±2.7^acCF^	4.38±2.9^aAC^	2.67±1.9^aCD^	2.93±1.5^aC^	2.88±1.5^aCE^	2.34±1.5^aCF^	2.19±1.2^aCF^
7d	5.95±2.9^aA^	4.18±1.8^aAB^	3.44±1.7^aAB^	2.98±1.7^adBC^	4.51±2.3^aAB^	2.38±1.1^aBC^	2.95±1.5^aB^	4.45±1.6^aAB^	2.04±1.0^aBC^	1.76±1.0^aBC^
14d	6.58±2.6^adA^	4.56±2.1^aABC^	5.04±3.0^aABC^	4.89±3.3^acdeABC^	4.48±2.7^aABC^	3.64±1.7^aABC^	3.95±2.0^aABc^	4.50±2.3^aABC^	4.03±1.8^aABC^	2.57±0.9^aBC^
21d	8.61±2.7^cdeBC^	5.33±2.7^aAC^	4.77±1.5^aA^	4.28±2.8^aeA^	4.52±3.8^aA^	4.06±2.1^aA^	4.23±1.2^aA^	4.27±3.5^aA^	2.59±1.7^aA^	2.74±1.6^aA^
28d	7.06±2.5^aeA^	4.36±2.5^aABC^	3.10±1.5^aB^	4.09±3.0^aeB^	4.58±2.1^aAB^	3.63±1.9^aB^	2.78±1.2^aB^	3.67±2.1^aB^	2.41±1.3^aB^	3.42±1.3^aB^

BA: bonding agent, CH: calcium hydroxide, d: day, DAB: double antibiotic paste, TAP: triple antibiotic paste, TAPM: modified triple antibiotic paste. Lowercase letters were used within the columns and uppercase letters within the rows for comparisons. Equal letters indicate statistic equivalence. Treatment*Period (p=0,007). For threshold comparisons, colors represent the level of specimen mismatch. Mismatches type A (ΔE_00_ raging between 1.8-3.6), mismatches type B (ΔE_00_ raging between 3.6-5.4), and mismatches type C (ΔE_00_ higher than 5.4) (Paravina et al., 2015) ^15^.

**Figure 1:**
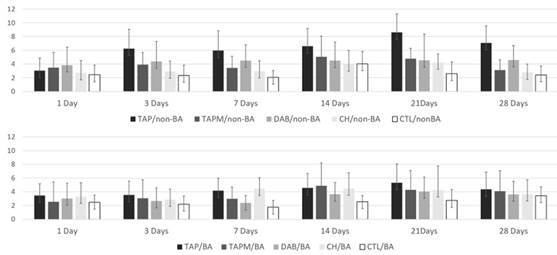
ΔE_00_ Means and standard deviations throughout the periods of evaluation (days).

Data analysis reveals that the TAP/non-BA group presented ∆E00 values similar to those from the other groups only on day 1. From day 3 to the end of the experiment at 28 days, TAP/non-BA presented the highest values of ∆E00 compared to the other groups (Figure 1).

The use of dentin bonding agents to seal the pulp chamber did not induce statistically significant differences in groups where TAPM, DAB, and CH pastes were used or in the control group (p>0,05). Only at day 21 the use of dentin bonding agent-induced significantly lower values of ∆E00 in the TAP group.

At the end of the experiment (days 21 and 28), the TAP/non-BA group presented the highest ∆E00 values. In contrast, CH/non-BA showed the lowest color variation within the experimental period.

In every experimental period, TAP/BA showed ∆E00 values similar to those from TAP/non-BA or the other evaluated groups. Additionally, the ∆E00 values from the TAP/BA group were lower than those from TAP/non-BA in every experimental period; however, this difference was insignificant except on day 21.

According to ∆E00 interpretive analysis, no group presented acceptable matches (∆E00<1.8). Considering dental discoloration as a frequent and undesired consequence of REP, this study set the category Mismatch type A (1.8-3.6) as a modest variation. In light of this, the results were present in three main categories: moderately unacceptable/tolerable effects (∆E00 ≤ 3,6), clearly unacceptable (mismatch type B, ∆E00 3,6-5,4), and highly unacceptable (mismatch type C, ∆E00 > 5,4) (Table 1). The TAP/non-BA group presented the worst results, classified as mismatch type C (∆E00>5.4) from day three onwards. At day 28, only TAPM/non-BA and CH/non-BA groups could be classified as type A mismatch, showing ∆E00 values lower than 3.6.

The control groups showed the best long-term chromatic stability in interpretative and statistical analysis. Therefore, CTL/non-BA and CTL/BA showed reliability in the type B mismatch category throughout the experiment.

**Figure 2:**
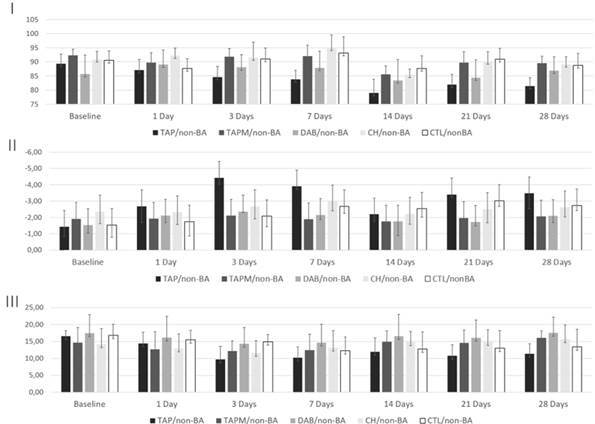
Variations of the coordinates: L* (lightness) (I); a* (red-green) (II); b* (yellow-blue) (III) in Non-bonded groups (non-BA).

**Figure 3:**
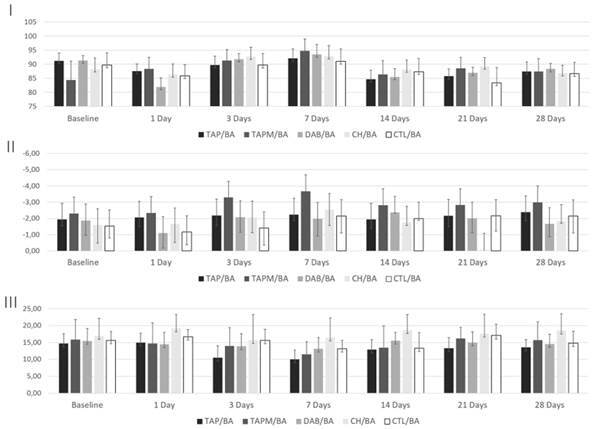
Variations of the coordinates: L* (lightness) (I); a* (red-green) (II); b* (yellow-blue) (III) in Bonded groups (BA).

After 28 days of storage, the specimens showed varied oscillations in the L* coordinate (lightness). The TAP/non-BA group samples presented the lowest values in the category. In general, specimens sealed with a dentin bonding agent (BA) showed higher lightness values than specimens from the non-bonding agent group (non-BA). The a* (green-red) coordinate presented results in the negative scale, indicating the presence of a green pigment, which increased over the evaluation periods (decrease in the a* axis). The b* axis, which indicates the yellow pigment, showed some stability along the experiment (slight alterations of the b* axis) (Figures 2 and 3).

The inclusion of Supplemental Tables1 and 2, which provide shades according to the VITA Classical A1-D4® Shade Guide, serves a specific purpose within the context of this study. These tables aim to create a link between the quantitative data derived from our precise colorimetric measurements and the more traditional, clinically familiar VITA shade guide that many clinicians use in their daily practice.

## Discussion

This study evaluated if sealing the pulp chamber with a dentin bonding agent could avoid tooth discoloration in REP. From the findings herein, it is possible to accept the first established hypothesis, in which the minocycline group (TAP) presents a higher potential for crown discoloration induction. The second established hypothesis that dentin bonding agents could reduce crow’s discoloration was partially accepted. Minocycline is an antibiotic agent in TAP’s composition, widely indicated for immature teeth root canal therapy ^12^ ^,^ ^17^. The suppression of minocycline in DAB or its replacement for another antibiotic in TAPM can represent a diminished antimicrobial potential of the pastes; therefore, alternative techniques are proposed to minimize the adverse effects on behalf of minocycline’s clinical efficacy ^11^ ^,^ ^18^ ^,^ ^19^.

Using bonding agents to seal the pulp chamber walls has been recommended. Since the dentinal tubules are obliterated, the medication cannot penetrate the dentin ^11^ ^,^ ^20^ ^,^ ^21^. The present study evaluated chromatic stability using the CIEDE2000 formula, which can detect small color changes ^22^ ^,^ ^23^. However, the role of dentin bonding agents in reducing color alteration was only evident considering the threshold comparisons.

Color-changing property of minocycline is caused by its interaction with dental tissues ^18^ ^,^ ^24^; however, this deleterious characteristic meets stabilization at a certain point. This was observed in TAP-non-BA and BA groups reaching their peak within 21 days. Remarkably, TAP groups (non-BA and BA) demonstrated the highest color variation among the groups, reaching extremely unacceptable results (mismatch type C) even in the initial evaluation periods (Day 3).

At day 14, the groups tend to stabilize ∆E00 values independently of bonding agent usage. On day 21, the groups reached a peak in color values, and the last period’s results (day 28) were similar to the previous period, confirming the stabilization of color changes.

The coordinates evaluation showed significant oscillation in the L* axis. The luminosity reduction, more prominent in non-bonding agent groups, possibly occurred due to the penetration of intracanal medications into the dentinal tubules ^11^ ^,^ ^16^. The a* coordinate presented values on a negative scale, characteristic of bovine teeth specimens. This behavior was also identified in previous studies with similar methods ^25^. Also, the slight variation spectrum of the b* pigment (yellow-blue) highlights that the perceived color changes were mainly due to the decrease in luminosity associated with the increasing greenish color.

Although REP presents the advantage of continuous root development, in thickness and length, with consequent increasing survival ratios of the tooth element, minocycline has a high potential to induce teeth discoloration, and the pulp chamber seal with dentin bonding agents cannot entirely avoid this unwanted event ^11^. Therefore, minocycline suppression (DAB) or replacement (TAPM) is the best alternative to prevent tooth discoloration in REP.

At present, some limitations of this study must be addressed. The antibiotic pastes are commonly obtained from local manipulation pharmacies and are not commercially available. In other words, the concentration of each component and the substances’ origin may differ by comparing various distributors. However, under the limitations of a laboratory study, our methods were designed to replicate procedures performed in clinics throughout the experimental phases to measure their impact on tooth color shade.

The potential of dentin bonding agents to safeguard against color changes post-Regenerative Endodontic Procedures (REP) holds significant clinical implications. This in vitro study highlights the importance of considering the color change after REP; and investigating the possible ways to avoid it, partially or entirely. Particularly noteworthy is the observation that teeth subjected to Triple Antibiotic Paste (TAP) manifested the most substantial chromatic variation. Still, the act of sealing the pulp chamber emerged as a potential protective measure, mitigating, albeit not fully warding off, the discoloration. The ΔE00 values, indicative of color shifts, were consistently deemed outside the acceptable clinical threshold, pointing predominantly to a lightness reduction and a shift toward green. The clinical significance herein pointed out is the prospect of a successful double scenario, which allows continuous radicular formation (REP main objective) and the diminished discoloration effects. While these findings offer valuable insights for endodontic practitioners, it's imperative to acknowledge the laboratory context of this study. Even though the results provide informative guidance, their translation to clinical settings necessitates careful consideration.

The seal of the pulp chamber with bonding agents reduces color dyschromia after REP using triple antibiotic paste. Besides, alternative pastes, such as TAPM and DAB, resulted in less dyschromia than traditional paste. Although the expected tooth discoloration occurs in the first weeks after the REP procedures, new studies should be developed to describe the long-term events possible to occur after REP. To our knowledge, this is the first laboratory study using bovine teeth and simulating clinical conditions using four of the most common medications applied in regenerative endodontics. This study performed high efforts to provide a standardized specimen preparation, emphasizing the behavior differences of each medication on the dental structure.

In conclusion, bonding agents did not significantly influence color alteration in DAB and CH groups, which performed better in their unsealed form. However, the pulp chamber seal reduced the dyschromia induced by TAP. Thus, considering bonding agents as an integral part of the dental professional's routine, their use can be recommended in cases where minocycline is necessary.

## Supplemental

**Table 1:** Color shades of non-bonded samples according to VITA Classical A1-D4® Shade Guide in 1, 14, and 28 days.

	TAP non-BA				TAPM non-BA				DAB non-BA				CH non-BA				Control non-BA		
SAMPLE	1D	14D	28D	SAMPLE	1D	14D	28D	SAMPLE	1D	14D	28D	SAMPLE	1D	14D	28D	SAMPLE	1D	14D	28D
1	A1	A1	A1	1	B1	B1	A1	1	B2	B2	A1	1	A1	B1	A1	1	A1	B1	A1
2	A1	B1	B1	2	B1	A1	A1	2	A1	B1	B1	2	A1	A1	A1	2	A1	A1	A1
3	A1	A1	B2	3	A1	A1	A1	3	A1	B1	A1	3	A1	A1	B2	3	B1	B1	B1
4	A1	A1	A1	4	A1	B1	A1	4	A1	B1	B2	4	B1	B1	B1	4	A1	A1	A1
5	A1	A1	A1	5	B1	A1	B1	5	A1	B1	A1	5	A1	B1	A1	5	A1	A1	A1
6	A1	A1	A1	6	A1	B1	A2	6	B1	A1	A1	6	B1	B1	B1	6	A1	A1	A1
7	A1	A1	D2	7	B1	B1	B1	7	A1	A1	B2	7	A1	A1	B2	7	B1	B1	B1
8	A1	B1	B1	8	A1	B1	A1	8	A2	A1	B2	8	B1	B1	B1	8	A1	B1	A1
9	A1	A1	A1	9	A1	B1	A1	9	A3,5	B4	A4	9	B1	B1	A1	9	A1	B1	B1
10	A1	A1	A1	10	A1	B1	A1	10	A1	A1	A1	10	A1	A1	A1	10	B1	B1	B1
11	B1	B1	A1	11	A1	A1	A1	11	B1	B1	B1	11	A1	B1	B1	11	A2	A1	A1
12	A1	A1	A1	12	A1	A1	A1	12	A3,5	B1	A1	12	B1	B1	B1	12	A1	B1	A1

**Supplemental Table 2:** Color shades of bonded samples according to VITA Classical A1-D4® Shade Guide in 1, 14, and 28 days.

	TAP BA				TAPM BA				DAB BA				CH BA				CTL BA		
SAMPLE	1D	14D	28D	SAMPLE	1D	14D	28D	SAMPLE	1D	14D	28D	SAMPLE	1D	14D	28D	SAMPLE	1D	14D	28D
1	B1	B1	A1	1	A1	B1	B1	1	B1	B1	B1	1	A1	A1	A3	1	A1	A1	C3
2	B1	B1	A1	2	A1	A1	A1	2	A1	B1	B1	2	A3	A1	B2	2	A1	A1	B1
3	A1	B1	A1	3	A1	B1	A1	3	A1	A1	A1	3	A1	A1	B1	3	B1	B1	A4
4	B1	B1	A1	4	A1	B1	B1	4	A1	A1	B2	4	A1	B1	A1	4	A1	A1	B2
5	A1	B1	B1	5	B1	B1	B1	5	B1	B1	A1	5	A1	B1	B1	5	A1	B1	A1
6	B1	B1	B1	6	B1	B1	A1	6	A1	B1	A1	6	B2	A1	A1	6	A1	B1	A1
7	B1	A1	A1	7	B2	B1	A1	7	A1	B1	A1	7	A1	A1	A1	7	A1	A1	A1
8	B1	B1	B1	8	A1	B1	A1	8	A1	B1	B1	8	A3	A3	B3	8	B1	B1	A1
9	B1	B1	B1	9	B1	B1	A1	9	A1	B1	A1	9	A1	A1	A1	9	B1	B1	A1
10	A1	B1	A1	10	B3	B1	A1	10	A1	B1	A1	10	B1	A1	A1	10	B1	B1	A1
11	A1	A1	A1	11	B1	B1	A1	11	A1	A1	B1	11	A1	B1	A1	11	A1	B1	A1
12	A1	B1	A1	12	A1	A1	A1	12	A1	A1	A1	12	B1	B1	B2	12	A1	A1	B1

TAP: Triple antibiotic paste, TAPM: Triple antibiotic modified paste, DAB: Double antibiotic paste, CH: Calcium hydroxide CTL: Control, BA: Bonding agent, D: Days.
